# Improving RNA Assembly via Safety and Completeness in Flow Decompositions

**DOI:** 10.1089/cmb.2022.0261

**Published:** 2022-12-13

**Authors:** Shahbaz Khan, Milla Kortelainen, Manuel Cáceres, Lucia Williams, Alexandru I. Tomescu

**Affiliations:** ^1^ Department of Computer Science and Engineering, IIT Roorkee, Roorkee, India.; ^2^ Department of Computer Science, University of Helsinki, Helsinki, Finland.; ^3^ School of Computing, Montana State University, Bozeman, Montana, USA.

**Keywords:** directed acyclic graphs, flow decomposition, flow networks, RNA assembly, safety

## Abstract

Decomposing a network flow into weighted paths is a problem with numerous applications, ranging from networking, transportation planning, to bioinformatics. In some applications we look for a decomposition that is optimal with respect to some property, such as the number of paths used, robustness to edge deletion, or length of the longest path. However, in many bioinformatic applications, we seek a specific decomposition where the paths correspond to some underlying data that generated the flow. In these cases, no optimization criteria guarantee the identification of the correct decomposition. Therefore, we propose to instead report the *safe* paths, which are subpaths of at least one path in every flow decomposition. In this work, we give the first *local* characterization of safe paths for flow decompositions in directed acyclic graphs, leading to a practical algorithm for finding the *complete* set of safe paths. In addition, we evaluate our algorithm on RNA transcript data sets against a trivial safe algorithm (extended unitigs), the recently proposed safe paths for path covers (TCBB 2021) and the popular heuristic *greedy-width*. On the one hand, we found that besides maintaining perfect precision, our safe and complete algorithm reports a significantly higher coverage (≈50% more) compared with the other safe algorithms. On the other hand, the greedy-width algorithm although reporting a better coverage, it also reports a significantly lower precision on complex graphs (for genes expressing a large number of transcripts). Overall, our safe and complete algorithm outperforms (by ≈20%) greedy-width on a unified metric (F-score) considering both coverage and precision when the evaluated data set has a significant number of complex graphs. Moreover, it also has a superior time (4−5×) and space performance (1.2−2.2×), resulting in a better and more practical approach for bioinformatic applications of flow decomposition.

## 1. INTRODUCTION

Network flows are a central topic in computer science, enabling us to define problems with countless practical applications. Assuming that the flow network has a unique source *s* and a unique sink *t*, every flow can be decomposed into a collection of weighted *s*–*t* paths and cycles (Ford and Fulkerson, 2010); for directed acyclic graphs (DAGs), such a decomposition contains only paths. One application of such a view of a flow is to indicate how to optimally route information or goods from *s* to *t*. For example, flow decomposition is a key step in network routing problems (Cohen et al, 2014; Hartman et al, 2012; Hong et al, 2013; Mumey et al, 2015) and transportation problems (Ohst, 2015; Olsen et al, 2022).

Finding the decomposition with the minimum number of paths and *possibly* cycles (or *minimum flow decomposition*) is NP-hard, even if the flow network is a DAG (Vatinlen et al, 2008). On the theoretical side, this hardness result led to research on approximation algorithms (Baier et al, 2005; Baier et al, 2002; Hartman et al, 2012; Mumey et al, 2015; Suppakitpaisarn, 2016; Pieńkosz and Kołtyś, 2015) and FPT algorithms (Kloster et al, 2018). On the practical side, many approaches usually use a standard *greedy-width* heuristic (Vatinlen et al, 2008), of repeatedly removing an *s*–*t* path carrying the most amount of flow. Another pseudopolynomial-time heuristic called *Catfish* was recently proposed by Shao and Kingsford (2017b), which tries to iteratively simplify the graph so that smaller decompositions can be found.

Many applications study flow networks built by superimposing a set of weighted paths, and seek the decomposition corresponding to that underlying set of paths and weights. This is the decomposition sought by the more recent and prominent application of reconstructing biological sequences [*RNA transcripts* (Bernard et al, 2013; Gatter and Stadler, 2019; Pertea et al, 2015; Tomescu et al, 2015; Tomescu et al, 2013; Williams et al, 2019) or *viral quasispecies genomes* (Baaijens et al, 2020; Baaijens et al, 2019)]. Each flow path represents a reconstructed sequence, and so, a different set of flow paths encode a different set of biological sequences, which may differ from the real ones.

If there are multiple flow decomposition solutions, then the reconstructed sequences may not match the original ones, and thus be incorrect. Williams et al (2021) analyzed an error-free transcript data set to find that 20% of the human genes admit multiple minimum flow decomposition solutions.

### 1.1. Overcoming multiple solutions

A first heuristic used to overcome the issue of multiple solutions (flow decompositions) was to seek one of minimum cardinality through different heuristics (Kloster et al, 2018; Pertea et al, 2015; Shao and Kingsford, 2017b; Tomescu et al, 2013) (since the problem is NP-hard). This approach was also used by transcript assemblers modeling the problem as a *minimum path cover* (Liu and Dickerson, 2017; Trapnell et al, 2010), which can be solved in polynomial time [see Cáceres et al (2021) for a comprehensive survey on the problem]. However, even when the solution is restricted to minimum cardinality, multiple solutions still arise (Caceres et al, 2021; Williams et al, 2021). Therefore, practical methods usually incorporate different variations of the minimum-cardinality criterion (Baaijens et al, 2020; Baaijens et al, 2019; Bernard et al, 2013).

Motivated by the RNA assembly application, Ma et al (2020a) were the first to address the issue of multiple solutions to the flow decomposition problem under a probabilistic framework. Later, they (Zheng et al, 2022) solved a problem (*AND-Quant*), which, in particular, leads to a quadratic-time algorithm for the following problem: given a flow in a DAG, and edges $e_{1},e_{2},\ldots ,e_{k}$, decide if in *every* flow decomposition there is always a decomposed flow path passing through all of $e_{1},e_{2},\ldots ,e_{k}$. Thus, by taking the edges $e_{1},e_{2},\ldots ,e_{k}$ to be the edges of a path *P*, the AND-Quant problem can decide if a path *P* (i.e., a given biological sequence) appears in all flow decompositions, which indicates that *P* is likely part of some original RNA transcript.

We build upon the AND-Quant problem, by addressing the flow decomposition problem under the *safety* framework (Tomescu and Medvedev, 2017), first introduced for genome assembly. For a problem admitting multiple solutions, a partial solution is said to be *safe* if it appears in all solutions to the problem. In the case of the flow decomposition problem, a path *P* is safe if for *every* flow decomposition into paths P, it holds that *P* is a subpath of some path in P. Furthermore, a path *P* is called *w-safe* if in *every* flow decomposition, *P* is a subpath of some weighted path(s) in P whose total weight is at least *w*. Safe paths for *all* flow decompositions are likely correct for many practical variations of the flow decomposition problem.

Safety has precursors in combinatorial optimization, under the name of *persistency*. For example, persistent edges present in all maximum bipartite matchings were studied by Costa (1994). Persistency has also been studied for the maximum flow problem, by finding that persistent edges always having a nonzero flow value in any maximum flow solution (Cechlárová and Lacko, 2001; Lacko, 1998), which is easily verified if the maximum flow decreases after removing the corresponding edge.

In bioinformatics, safety has been previously studied for the genome assembly problem, which at its core solves the problem of computing arc-covering walks on the assembly graph. Again since the problem admits multiple solutions where only one is correct, practical genome assemblers output only those solutions likely to be correct. The prominent approach dating back to 1995 (Kececioglu and Myers, 1995) is to compute trivially correct *unitigs* (having internal nodes with *unit* indegree and unit outdegree), which can be computed in linear time. Later, unitigs were generalized to be *extended* by adding their unique incoming and outgoing paths (Jackson, 2009; Kingsford et al, 2010; Medvedev et al, 2007; Pevzner et al, 2001).

These *extended unitigs*, although safe, are not guaranteed to report *everything* that can be correctly assembled, presenting an important open question (Boisvert et al, 2010; Bresler et al, 2013; Guénoche, 1992; Lam et al, 2014; Nagarajan and Pop, 2009; Shomorony et al, 2016) about the *assembly limit* (if any). This question was finally resolved by Tomescu and Medvedev (2017) [later optimized in Cairo et al (2021) and Cairo et al (2019)] for a specific genome assembly formulation (single circular walk) by introducing *safe and complete* algorithms, which report everything that can be theoretically reported as safe. Safe and complete algorithms were also studied by Acosta et al (2018) under a different genome assembly formulation of multiple circular walks.

Recently, Caceres et al (2021) studied safe and complete algorithms for path covers in an application on RNA assembly. They optimized an *avoid-and-test* approach for computing all maximal safe paths for *constrained path covers*, which were able to cover 70% of transcripts with a precision of more than 99% on splice graphs built from transcript annotation.

### 1.2. Flow decomposition in RNA assembly

The prominent application of flow decomposition in bioinformatics is the RNA transcript assembly, which is described as follows. In complex organisms, a gene may produce multiple RNA molecules (*RNA transcripts*, i.e., strings over an alphabet of four characters), each having a different abundance. Current *high-throughput sequencing* techniques (Wang et al, 2009) allow to partially read the RNA transcripts (and find their abundances) from a sample. This technology produces short overlapping substrings of the RNA transcripts. The main approach for recovering the RNA transcripts from such data is to build an edge-weighted DAG from these fragments, then to transform the weights into flow values by various optimization criteria, and finally to decompose the resulting flow into an “optimal” set of weighted paths (i.e., the RNA transcripts and their abundances in the sample) (Mäkinen et al, 2015).

A common approach used in practice is the popular *greedy-width* heuristic (Pertea et al, 2015; Tomescu et al, 2013). Greedy-width is also used in the related problem of viral quasispecies assembly (Baaijens et al, 2019; Fritz et al, 2021). Furthermore, some tools attempt to incorporate additional information into the flow decomposition process, such as by using longer reads or super reads (Gatter and Stadler, 2019; Pertea et al, 2015; Shao and Kingsford, 2017a; Williams et al, 2021). Despite the large number of tools and methods that have been developed for RNA transcript assembly, there is no method that consistently reports the correct set of transcripts (Pertea et al, 2015; Yu et al, 2020). All these, plus the promising results of safe and complete algorithms for constrained path covers (Caceres et al, 2021), suggest that addressing the problem under the safety framework may be a promising approach. However, while a safe and complete solution clearly gives the maximally reportable correct solution, it is significant to evaluate whether such a solution covers a large part of the true transcript, to be useful in practice. A possible application of such partial and reliable solutions is to consider them as constrains [see e.g., Williams et al (2021)] of real RNA transcript assemblers, to guide the assembly process of such heuristics. Another possible application could be to evaluate the accuracy of assemblers: does the output of the assembler include the safe and complete solution?

### 1.3. Our results

Our contributions can be succinctly described as follows.

1. **A simple local characterization resulting in an optimal verification algorithm:** We give a characterization for a safe path *P* using its local property called *excess flow*.

**Theorem 1.** *For* w>0, *a path P is w*-*safe iff its excess flow* $f_{P}\geq w$.

The previous work (Zheng et al, 2022) on AND-Quant describes a *global* characterization using the maximum flow of the entire graph transformed according to *P*, requiring O(mn) time. Instead, the excess flow is a *local* property of *P* computable in time linear on its length. This also directly gives a simple and optimal verification algorithm.

**Theorem 2.** *Given a flow graph (DAG) having n vertices and m edges, it can be preprocessed in* O(m) *time to verify the safety of any path P in* O(|P|)=O(n) *time.*

2. **Simple enumeration algorithm:** The previous characterization also results in a simple algorithm for reporting all maximal safe paths by using an arbitrary flow decomposition of the graph.

**Theorem 3.** *Given the paths* P *in a flow decomposition, all its maximal safe paths can be reported in* O(||P||) *time.*

This approach starts with a candidate solution and uses the characterization on its subpaths in an efficient manner [a similar approach was previously used by Costa (1994); Acosta et al (2018); Caceres et al (2021)]. In particular, since O(mn)-sized flow decomposition can be computed in O(mn) time (Ahuja et al, 1993), we obtain the following corollary.

**Corollary 1.** *Given a flow graph (DAG) with n vertices and m edges, all its maximal safe paths can be reported in time* O(mn).

The solution of the algorithm is reported using a compact representation (referred as $P_{c}$), whose size can be Ω(mn) in the worst case, but merely O(m+n) in the best case.

3. **Empirically improved approach for RNA assembly:** Using simulated RNA splice graphs, we found that safe and complete paths for flow decompositions provide precise RNA assemblies while covering most of the RNA transcripts. Safe and complete paths are ≈50% better in coverage over previous notions of safe paths, while maintaining the perfect precision ensured by safety. Furthermore, for the combined metric for coverage and precision (F-score), the safe and complete paths outperform the popularly used greedy-width heuristic significantly (≈20%) and the previous safety algorithms appreciably (≈13%). Finally, although our approach takes 1.2−2.5× time than the trivial safe algorithms requiring equivalent memory, the greedy-width approach requires roughly 4−5× time and 1.2−2.2× memory, while the safe paths for path covers are outperformed by one order of magnitude.

Hence, the significance of our approach in quality parameters increases with the increase in complex graph instances in the data set, while the performance parameters are significantly better than greedy-width, without significantly losing performance over the previous safe algorithms.

## 2. PRELIMINARIES AND NOTATIONS

We consider a DAG G=(V,E) with *n* vertices and *m* edges, where each edge *e* has a positive flow f(e) passing through it (also called its *weight*). Without loss of generality, we assume the graph is connected, and hence m≥n. We assume that *G* contains a unique source with no incoming edges, denoted as *s*, and a unique sink with no outgoing edges, denoted as *t*. As such, $f_{in}\left(s\right)=f_{out}\left(t\right)=0$. Every other vertex *v* satisfies the *conservation of flow* $f_{in}\left(v\right)=f_{out}\left(v\right)$, making the graph a *flow graph*. For a path *P* in the graph, |P| denotes the number of its edges. For a set of paths $P=\left\{P_{1},\ldots ,P_{k}\right\}$, we denote its total size (number of edges) by $\left|P|=|P_{1}|+\ldots +|P_{k}\right|$.

For any flow graph (DAG), a *flow decomposition* of it, it is a set of weighted *paths* P such that the flow on each edge of the flow graph equals the sum of the weights of the paths containing the edge. It is well known (Ahuja et al, 1993) that a flow decomposition P of at most *m* paths can be computed in time O(||P||)=O(mn). A simple algorithm takes at each step the smallest flow edge and extends it to *s* and *t*. A path *P* is called *w-safe* if, in every possible flow decomposition, *P* is a subpath of some paths in $P_{f}$ whose total weight is at least *w*. If *P* is *w*-safe with w>0, we call *P* a *safe flow path*, or simply a *safe path*. Intuitively, for any edge *e* with nonzero flow, we consider *where did the flow on e come from?*

**FIG. 1.**
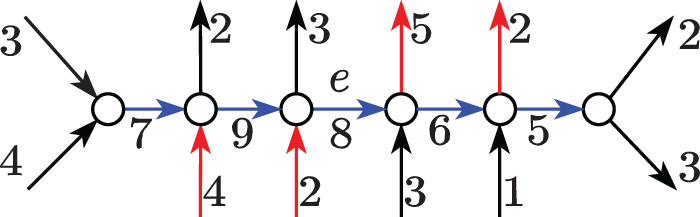
The prefix of the path (*blue*) up to *e* contributes at least 2 U of flow to *e*, as the rest may enter the path by the edges (*red*) with flow 4 and 2. Similarly, the suffix of the path (*blue*) from *e* maintains at least 1 U of flow from *e*, as the rest may exit the path from the edges (*red*) with flow 5 and 2. Both these safe paths are *maximal* as they cannot be extended left or right.

We would like to report all the maximal paths ending with *e* along which some w>0 weight always “flows” to *e* (see Fig. 1). A safe path is *left maximal* (or *right maximal*) if extending it to the left (or right) with any edge makes it unsafe (i.e., not safe). A safe path is *maximal* if it is both left and right maximal. A set of safe paths is called *complete* if it consists of *all* the maximal safe paths.

Previous notions of safety used in other problems naturally extend to flow decomposition as follows. Paths having internal nodes with unit indegree and unit outdegree are called *unitigs* (Kececioglu and Myers, 1995). Unitigs are trivially safe because every source-to-sink path passing through an edge of a unitig also passes through the entire unitig. Furthermore, a unitig can naturally be *extended* to include its unique incoming path (having nodes with unit indegree), and its unique outgoing path (having nodes with unit outdegree). This extension of a unitig is called the *extended unitig* (Jackson, 2009; Kingsford et al, 2010; Medvedev et al, 2007; Pevzner et al, 2001), which is also safe using the same argument.

For some graphs the above notions already define the safety of flow decomposition *completely*. Recently, Millani et al (2020) defined a class of DAGs called *funnels*, where every source-to-sink path is uniquely identifiable by at least one edge, which is not used by any other source-to-sink path. Hence, considering such an edge as a trivial unitig (having a single edge), its extended unitig is exactly the corresponding source-to-sink path, making it safe. Thus, in a funnel, all source-to-sink paths are naturally safe and hence trivially complete. Moreover, it implies that a funnel has a unique flow decomposition, making the problem trivial for funnel instances.

**Theorem 4.** *For a DAG G, G is a funnel iff the set of extended unitigs of G is safe and complete.*

*Proof.* The forward direction of the equivalence was already discussed. For the reverse direction suppose by contradiction that *G* is not a funnel. Consider a minimal butterfly subgraph of *G*. As such, the body of this butterfly is a unitig in *G*. Since the first vertex of this unitig has an indegree greater than 1, and the last vertex of the unitig has an outdegree greater than 1, it is also an extended unitig. However, this path is not left or right maximal. Therefore, the set of maximal safe paths is not complete, a contradiction.

Finally, since flow decompositions are always constrained path covers, safe and complete paths for constrained path covers (Caceres et al, 2021) are potentially not complete for flow decompositions. The main hypothesis of our work is that the maximal safe paths for flow decompositions are significantly longer than the previous notions of safety for RNA transcript assembly.

## 3. CHARACTERIZATION AND PROPERTIES OF SAFE AND COMPLETE PATHS

**FIG. 2.**
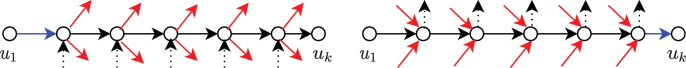
The excess flow of a path (left) is the incoming flow (*blue*) that necessarily passes through the whole path despite the flow (*red*) leaving the path at its internal vertices. It can be analogously described (right) as the outgoing flow (*blue*) that necessarily came through the whole path despite the flow (*red*) entering the path at its internal vertices.

The safety of a path can be characterized by its *excess flow* (see Fig. 2), defined as follows.

**Definition 1** (Excess flow). *The* excess flow *f_P_ of a path* $P=u_{1},u_{2},\ldots ,u_{k}$ *is*

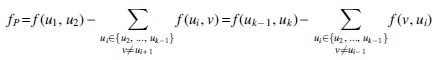

*where the former and later equations are called diverging and* converging *formulations, respectively.*

**Remark 1.** *Alternatively, the converging and diverging formulations can be described as*

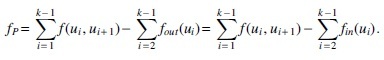

The converging and diverging formulations are equivalent by the conservation of flow on internal vertices. The idea behind the notion of an excess flow *f_P_* is that even in the worst case, the maximum *leakage* (see Fig. 2), that is, the flow leaving (or entering) *P* at the internal nodes, is the sum of the flow on the outgoing (or incoming) edges of the internal nodes of *P*, that are not in *P*. However, if the value of incoming flow (or outgoing flow) is higher than this maximum leakage, then this excess value *f_P_* necessarily passes through the entire *P*. The following results give the simple characterization and an additional property of safe paths.

**Theorem 1.** *For w* > 0, *a path P is w-safe iff its excess flow f_P_* ≥ *w*.

*Proof.* The excess flow *f_P_* of a path *P* trivially makes it $w\leq f_{P}$-safe by definition. If $f_{P}<w$, we can prove that *P* is not *w*-safe by modifying any flow decomposition having *w* flow on *P* to leave only *f_P_* flow (or 0, if $f_{P}<0$) on *P* as follows. In Figure 2 (diverging), consider a flow path P′ entering *P* through edge *e*_1_ [except first edge (blue)] and leaving *P* at an edge $e_{2}\left(red\right)$ except the last edge of *P*. Since $f_{P}<w$, it is not possible that every path leaving *P* using a red edge starts at the first blue edge (by definition of *f_P_*), hence P′ always exists. We modify P′ by using flow on *P* to form two paths, which enter from *e*_1_ and leave at the last edge, and which enter from the first edge and leave at *e*_2_.

We can repeat such modifications until flow on *P* is *f_P_* (or 0, if $f_{P}<0$) due to the conservation of flow. In addition, for a path to be safe, it must hold that w>0.

**Lemma 1.** *For any path in a flow graph (DAG), adding an edge* (u,v) *to its start or its end reduces its excess flow by* $f_{in}\left(v\right)-f\left(u,v\right)$*, or* $f_{out}\left(u\right)-f\left(u,v\right)$*, respectively.*

*Proof.* Using the converging formulation in Remark 1, adding an edge at the start of a path modifies its excess flow by $f\left(u,v\right)-f_{in}\left(v\right)$. Similarly, using the diverging formulation in Remark 1, adding an edge at the end of a path modifies its excess flow by $f\left(u,v\right)-f_{out}\left(u\right)$.

## 4. SIMPLE VERIFICATION AND ENUMERATION ALGORITHMS

### 4.1. Verification algorithm

The characterization (Theorem 1) can be directly adapted to verify the safety of a path optimally. We preprocess the graph to compute the incoming flow $f_{in}\left(u\right)$ and outgoing flow $f_{out}\left(u\right)$ for each vertex *u* in total O(m) time. Using Remark 1 the time taken to verify the safety of any path *P* is O(|P|)=O(n), resulting in the following theorem.

**Theorem 2.** *Given a flow graph (DAG) having n vertices and m edges, it can be preprocessed in O(m) time to verify the safety of any path P in O(|P|) = O(n) time.*

### 4.2. Enumeration of all maximal safe paths

Given a flow decomposition P, we show how to report all the maximal safe paths. To do so, we adapt the approach of Caceres et al (2021) for the same task on constrained path covers. They run a two-pointer algorithm on each path P∈P (in their case P is a constrained path cover) to find maximal safe paths on *P*.

The main idea of the algorithm is that it is possible to use two pointers to vertices delimiting a subpath P′ of *P*, which is tested for safety. Since only *maximal* safe paths are required, the algorithm extends the right pointer as much as possible, while still being safe. If no further (right) extension is possible, then it advances the left pointer (and also the right if they are in the same position), and repeats. Because the left pointer never surpasses the right pointer, the algorithm takes O(|P|) safety tests for P∈P, O(||P||) in total.

Caceres et al (2021) (Lemma 3) implement a safety test taking O(|P|m) time, obtaining the O(|P|m||P||) time solution. In our case we can use Theorem 2 to test the safety of a path in linear time, automatically deriving the O(|P′|⋅||P||)=O(n||P||) time algorithm. Moreover, we note that we can update the excess flow of a path in constant time when moving one of the pointers during the algorithm (see Lemma 1). However, reporting a safe path P′ still takes O(|P′|) time. We solve this by instead reporting the two pointers representing P′.

**Theorem 3.** *Given the paths* P *in a flow decomposition, all its maximal safe paths can be reported in* O(||P||) *time*.

**Concise representation:** The solution can be reported using a concise representation (referred as $P_{c}$) having a set of paths as follows. We add to $P_{c}$ every subpath of each path P∈P that contains maximal safe paths, along with the indices of the solution on the path. Thus, for one or more overlapping maximal safe subpaths from *P*, we add a single path in $P_{c}$, which is the union of all such maximal safe paths, making the paths added to $P_{c}$ of *minimal* length. Finally, we also remove the duplicates and prefixes/suffixes among the maximal safe subpaths reported from different paths in P using an Aho Corasick trie (Aho and Corasick, 1975), making the set of paths in $P_{c}$ *minimal*. Thus, we define $P_{c}$ as follows.

**Definition 2** (Concise representation $P_{c}$). *A minimal set of paths having a minimal length such that every safe path of the flow network is a subpath of some path in the set*.

**Remark 2.** *In the worst case, the algorithm is optimal for DAGs having* $|P_{c}|=|P|=\Omega \left(mn\right)$*, but in general* $\left|P_{c}\right|$ *can be as small as* O(m+n) *(see the next section). Thus, improving this bound requires us to not use a flow decomposition (and hence a candidate solution).*

### 4.3. Tightness and worst case for a simple enumeration algorithm

**FIG. 3.**
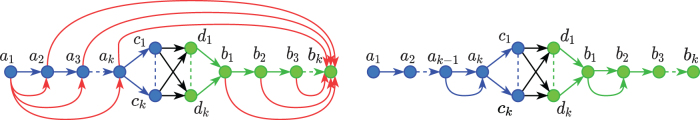
The worst case (left) and best case (right) graphs for the simple enumeration algorithm. **(a)** Catfish data set, **(b)** Reference-Sim data set.

The example shown in Figure 3 demonstrates the worst case and the best case graphs where the simple enumeration algorithm is optimal, and inefficient, respectively. We have two paths $A=\left\{a_{1},\ldots ,a_{k}\right\}$ and $B=\left\{b_{1},\ldots ,b_{k}\right\}$. The set $C=\left\{c_{1},\ldots ,c_{k}\right\}$ has edges from *a_k_* and the set $D=\left\{d_{1},\ldots ,d_{k}\right\}$ has edges to *b*_1_. Choosing k=n∕4 and any subset of connections between C×D, we get a graph with any *n* and *m*. Let there be flow *k* on the black edges and unit flow on the red edges.

(1) In the worst case graph (left), the flow on the remaining edges is according to the flow conservation assuming *a*_1_ as the source and *b_k_* as the sink. Each edge in C×D necessarily has a separate path in P from *a*_1_ to *b_k_*, with *k* maximal safe paths between $\left\{a_{i},b_{i}\right\}$ for all 1≤i≤k because every path between *a_i_* to *b*_1_ has excess flow *i*. This ensures that $|P_{c}|=|P|=\Omega \left(mn\right)$.

(2) In the best case graph (right), the two edges from $a_{k-1}$ to *a_k_* and from *b*_1_ to *b*_2_ carry equal flow, and the remaining edges have flow according to the conservation of flow. Each edge in C×D has a safe path of O(1) size from *a_k_* to *b*_1_. In addition, there are two safe paths each of length O(n) from *a*_1_ to *a_k_*, and from *b*_1_ to *b_k_*, corresponding to two parallel edges between $\left(a_{k-1},a_{k}\right)$, and between $\left(b_{1},b_{2}\right)$, respectively. However, we still have |P|=Ω(mn) but $|P_{c}|=O\left(m+n\right)$.

## 5. EXPERIMENTAL EVALUATION

We now evaluate the performance of our safe and complete algorithm on the problem of RNA Assembly. We consider the flow networks representing splice graphs of simulated RNA-Seq experiments. That is, starting from a set of RNA transcripts, we simulate their expression levels and superimpose the transcripts to create a flow graph. Evaluating our approach in such perfect scenario allows us to remove the biases introduced by real RNA-Seq experiments (Srivastava et al, 2020) and focus the features offered by each technique instead. We say that the number *k* of transcripts or ground truth paths^*^ is the complexity of the graph. Intuitively, the more paths in the ground truth, the harder to decompose the corresponding splice graph.

We first investigate the practical significance of safety by comparing our solution with the popular flow decomposition heuristic greedy-width. Greedy-width (Vatinlen et al, 2008) decomposes the flow by sequentially selecting the heaviest possible path, resulting in a simple algorithm that is both scalable and performs well in practice. However, flow decomposition algorithms may not always report the ground truth paths, but a different (incorrect) solution. Thus, it is important to measure the reported solution using a precision metric that evaluates the correctness of the solution. We thus investigate how the precision of greedy-width varies particularly as the value of *k* increases.

We then investigate the practical significance of completeness as reported by our solution, over the previously known safe solutions as reported by extended unitigs (recall Section 2) and safe paths for path covers of Caceres et al (2021) (recall Section 4.2). Note that every safe solution should be 100% precise by definition.^†^ Hence, all safe solutions should always outperform greedy-width (or any flow decomposition algorithm) in terms of precision. However, this perfect precision comes at the cost of a smaller reported solution. Intuitively, this can be measured using some coverage metrics describing how much of the ground truth sequence is included in the reported paths. We investigate how the coverage of the different safe solution varies with respect to greedy-width, particularly as the value of *k* (complexity of the graph) increases.

Finally, to understand the overall impact of the different approaches, we combine the coverage and precision measures by computing their harmonic mean, that is, F-score.^‡^ We thus investigate the variation in F-score over different values of *k* (graph complexities). In addition, to understand the practicality of the algorithms, we also measure their time and space performance.

### 5.1. Data sets

We consider two RNA transcript data sets, generated based on the approach of Shao and Kingsford (2017b). They created “perfect” flow graphs where the true set of transcripts and abundances is always a flow decomposition of the graph (which also means that the graphs satisfy conservation of flow). They start with a ground truth set of transcripts and abundances and create the input instances by superimposing these transcripts into a single flow graph with a unique source *s* pointing to the beginning of each transcript, and a unique sink *t* pointed from the end of each transcript.

#### 5.1.1. Funnel instances

As described in Section 2, funnels (Millani et al, 2020) have a unique flow decomposition, thus making the problem trivial. As such, any flow decomposition algorithm (including greedy-width) reaches perfect scores in coverage and precision on these instances. Moreover, as shown in Theorem 4, this is also the case for extended unitigs, and thus for our safe and complete algorithm. Interestingly, safe paths for constrained path covers (Caceres et al, 2021) are not necessarily complete on funnels.^§^ This means that for any flow decomposition algorithm (including greedy-width) and most safe algorithms (including extended unitigs and our safe and complete algorithm), the resulting paths always achieve the perfect value of coverage, precision, and F-score on funnel instances. As a result, funnels dilute the relative measures of the different algorithms.

**FIG. 4.**
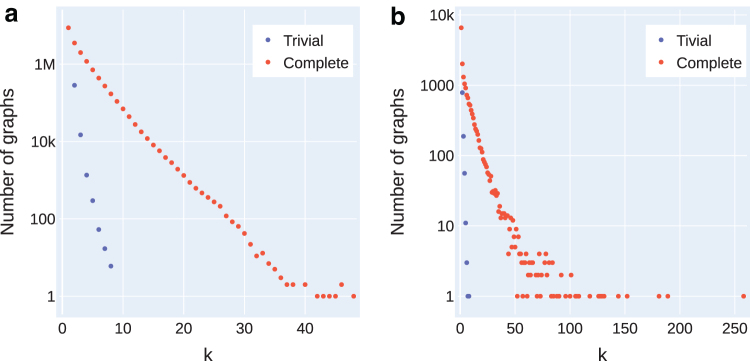
Distribution of graphs in the data sets by its complexity *k* with respect to the trivial instances (funnels). **(a)** Catfish dataset and **(b)** Reference-Sim dataset.

Previously, Kloster et al (2018, Lemma 8) described a contraction of graphs that transforms funnels to trivial instances (k=1), however, they excluded only single-path instances from their evaluation. We found (see Fig. 4) that many complex instances (with larger *k*) are also funnels. Hence, we removed such instances from our evaluation for a more accurate presentation of our results. Since the previous studies (Kloster et al, 2018; Shao and Kingsford, 2017b; Williams et al, 2021) have considered the complete data sets including the trivial instances, we also include the evaluation on the complete data sets for the sake of completeness.

#### 5.1.2. Catfish data set

We consider the data set first used by Shao and Kingsford (2017b), which includes 100 simulated human transcriptomes for human, mouse, and zebrafish built using the Flux-Simulator (Griebel et al, 2012). In addition, it includes 1000 experiments from the Sequence Read Archive, with simulated abundances for transcripts using salmon (Patro et al, 2017). In both cases, the weighted transcripts are superimposed to build splice graphs as described above. This data set has also been used in other flow decomposition benchmarking studies (Kloster et al, 2018; Williams et al, 2021). There are 17,335,407 graphs in total in this data set, of which 8,301,682 are nontrivial (47.89%). The log-scale distribution of the complete data set (and its funnels) by *k* is shown in Figure 4a. However, in this data set, the details about the number of bases on each node (exons or pseudoexons) are omitted, only allowing us to compute the metrics in terms of nodes.

#### 5.1.3. Reference-Sim data set

We modified the procedure described for building the splice graphs of the human transcriptome by Caceres et al (2021). For each transcript, we first simulate its expression level by sampling a value from the lognormal distribution^**^ with mean and variance both equal to −4, as done in the default settings of the RNASeqReadSimulator (Li, 2014). Finally, we compute the corresponding flow on the splice graphs by superimposing the different transcripts with their corresponding abundances. There are 17,941 total graphs in this data set, of which 10,323 are nontrivial (57.54%). The log-scale distribution of the complete data set (and its funnels) by *k* is shown in Figure 4b. In this data set, we also have access to the genomic coordinates (and hence the number of bases) represented by nodes, allowing us to also compute the metrics in terms of bases.

### 5.2. Evaluation metrics

We compute the metric weighted precision, maximum relative coverage as done by Caceres et al (2021). In addition, we compute F-score as the harmonic mean of the other two. For the sake of completeness we explain them next.

#### 5.2.1. Weighted precision

We classify a reported path *R* as correct if *R* is a subpath of some ground truth transcript *T* of the flow graph. Weighted precision is the total length of correctly reported paths divided by the total length of reported paths. The commonly used precision metric (Pertea et al, 2015; Shao and Kingsford, 2017a) for measuring the accuracy of RNA assembly methods considers only as correct those paths that are (almost) exactly contained in the ground truth decomposition, and precision is computed as the number of correctly reported paths divided by the total reported paths. However, since the safe algorithms report (possibly) partial transcripts, we use subpaths instead of (almost) exactly the same paths. To highlight how much is reported correctly instead of how many, we use weighted precision to give a better score for longer correctly reported paths.

#### 5.2.2. Maximum relative coverage

Given a ground truth transcript *T* and a reported path *R*, we define a segment of *R* inside *T* as a maximal subpath of *R* that is also a subpath of *T*. We define the maximum relative coverage of *T* as the length of the longest segment of a reported path inside *T*, divided by the length of *T*. The corresponding value for the entire graph is the average of the values over all transcripts of the graph. While it is common in the literature (Pertea et al, 2015; Shao and Kingsford, 2017a) to report sensitivity (the proportion of ground truth transcripts correctly predicted), we measure sensitivity based on coverage since the safe algorithms report paths that (possibly) do not cover an entire transcript.

#### 5.2.3. F-score

The standard measure to combine precision and sensitivity is using an F-score, which is the harmonic mean of the two. In our evaluation, we correspondingly use the weighted precision and the maximum relative coverage for computing the F-score.

We compute all metrics in terms of nodes and bases for the Reference-Sim data set. For the Catfish data sets, we only report them in terms of nodes.

### 5.3. Implementation and environment details

We evaluate the following approaches in our experiments.

#### 5.3.1. SafePC

It computes the safe paths for path covers by using the implementation of Caceres et al (2021). We use S={s}, T={t}, and ℓ=k+1 as recommended by the authors (Caceres et al, 2021; Section 3.1).

#### 5.3.2. ExtUnitigs

It computes the extended unitigs, by considering each unitig including single edges, and extending it toward the left as long as the internal nodes have unit indegree, and toward the right as long as internal nodes have unit outdegree.

#### 5.3.3. Safe&Comp

It computes the safe and complete paths for flow decomposition using our enumeration algorithm described in Section 4. However, since the metric evaluation scripts use each safe path individually (as reported by other algorithms), we output all safe paths separately instead of using $P_{c}$. This increases the size of output and hence the time complexity to $O\left(mn^{2}\right)$ from O(mn) as stated in Theorem 1.

#### 5.3.4. Greedy

It computes the greedy-width heuristic using Catfish (Shao and Kingsford, 2017b) with the -a greedy parameter.

All algorithms are implemented in C++, whereas the scripts for evaluating metrics are implemented in Python. SafePC, ExtUnitigs, and Safe&Comp implementations use optimization level 3 of GNU C++ (compiled with −O3 flag), whereas the Greedy uses the optimizations of the Catfish pipeline. SafePC, ExtUnitigs and Safe&Comp additionally require a postprocessing step for removing duplicates, and prefix/suffixes, to make the set of safe paths minimal. However, the time and memory requirements are evaluated considering only the algorithm, and not postprocessing and metric evaluations. All experiments were evaluated on a laptop using a single core (i7-8750H CPU 2.2 GHZ) having 16GB memory. The source code of our project is available on GitHub^††^ under GNU General Public License v3.

### 5.4. Results

**FIG. 5.**
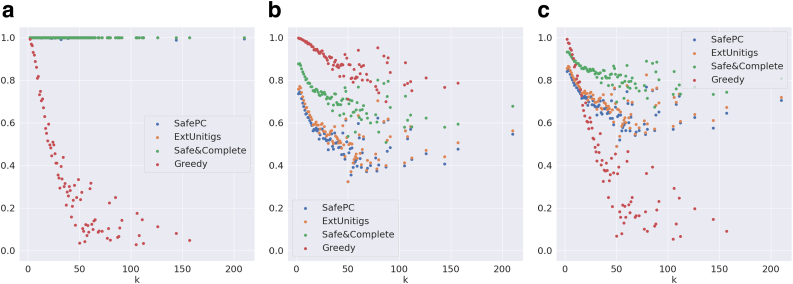
Evaluation metrics on graphs distributed by *k* for the Reference-Sim data set. **(a)** Weighted precision, **(b)** maximum coverage, and **(c)** F-score.

We first evaluate the significance of safety among the reported solutions. Figure 5a compares the weighted precision, distributed over the complexity *k* (number of transcripts in the ground truth), of all the algorithms on the Reference-Sim data set. Safe algorithms (except SafePC) report perfect precision as expected. In the case of SafePC, there is a small loss in precision due to the pruning of solutions performed by the algorithm. However, the precision of the Greedy algorithm sharply declines with the increase in *k*, almost linearly to 30% for k=35. This may be explained by the sharp increase in the number of possible paths in complex graphs, hindering the task to any flow decomposition algorithm. Hence, the significance of safety becomes very prominent as *k* increases.

Next, we evaluate the significance of completeness of the safe algorithms. Figure 5b compares the maximum relative coverage, distributed over *k*, of all the algorithms on the Reference-Sim data set. As expected, Greedy outperforms all the other, followed by Safe&Comp, ExtUnitigs, and SafePC. The reason why ExtUnitigs outperforms SafePC is because the latter only requires to cover the nodes of the splice graph, motivating further research on the same techniques, but for edge path covers. We note that, as *k* reaches 20, Safe&Comp, ExtUnitigs, and SafePC sharply fall to 75%, 60%, and ∼60% respectively, while Greedy maintains around 95% coverage. Overall, Safe&Comp is almost always ≈85%−90% of that of Greedy, whereas ExtUnitigs and SafePC fall to 60%. Hence, Safe&Comp manages to maintain perfect precision without losing a lot on coverage, demonstrating its dominance on completeness among the safe algorithms.

Figure 5c supports the above inference by evaluating the combined metric F-score. Safe&Comp dominates SafePC and ExtUnitigs by definition. Safe&Comp also dominates Greedy for k>10. It is also important to note that both ExtUnitigs and SafePC eventually dominate Greedy for a slightly larger value of k>20. This shows the significance of considering safe algorithms for complex graphs.

Besides, we evaluate a summary of the above results averaged over all graphs regardless of *k*. Table 1 summarizes the evaluation metrics of all the algorithms for simple graphs (k≤10) and complex graphs (k>10), and both. While on simpler graphs Greedy dominates Safe&Comp mildly (≈3%), for complex graphs it is dominated significantly (≈20%) by Safe&Comp and appreciably (≈8%) by ExtUnitigs. However, despite the larger ratio of simple graphs, the collective F-score over all graphs is still (≈4%) better for Safe&Comp over Greedy.

**Table 1.** Summary of Evaluation Metrics for the Reference-Sim Data Set

Graphs	Algorithm	Maximum coverage	Weighted precision	F-score
k≥2 (100%)	SafePC	0.66	1.00	0.79
	ExtUnitigs	0.69	1.00	0.81
	Safe&Comp	0.82	1.00	0.90
	Greedy	0.98	0.81	0.86
2≤k≤10 (68%)	SafePC	0.70	1.00	0.82
	ExtUnitigs	0.73	1.00	0.84
	Safe&Comp	0.84	1.00	0.91
	Greedy	0.99	0.91	0.94
k>10 (32%)	SafePC	0.58	1.00	0.73
	ExtUnitigs	0.61	1.00	0.75
	Safe&Comp	0.76	1.00	0.86
	Greedy	0.95	0.60	0.69

Finally, we evaluate the practicality of the algorithms by comparing their running time and peak memory. In Tables 2 and 3, we see that ExtUnitigs are the fastest, whereas Safe&Comp takes roughly 1.2−3× time than ExtUnitigs, and Greedy requires roughly 4−5× time than Safe&Comp. In the case of SafePC, its more complex verification algorithm worsens its running time by roughly one order of magnitude compared with the other safe approaches. The peak memory of the ExtUnitigs and Safe&Comp is very close (within 5%–25%), whereas Greedy and SafePC require roughly 1.3−3.6× and 1.3−6.3 more memory than Safe&Comp, respectively. Overall, in terms of time/space performance, Safe&Comp shows a significant improvement over Greedy, without losing a lot over the trivial algorithm.

**Table 2.** Time and Memory Requirements of the Different Algorithms for the Evaluated Data Sets

Algorithm	Reference-Sim		Catfish			
	Human 25.6 MB		Zebrafish 122 MB		Mouse 137 MB	
	Time (seconds)	Memory (MB)	Time (seconds)	Memory (MB)	Time (seconds)	Memory (MB)
SafePC	10.64	3.74	144.75	10.93	146.46	10.10
ExtUnitigs	0.21	2.95	11.02	3.07	9.79	3.07
Safe&Comp	0.65	3.01	14.83	2.96	14.24	3.16
Greedy	2.78	3.85	74.00	7.84	75.87	5.64

**Table 3.** Time and Memory Requirements of the Different Algorithms for the Evaluated Data Sets (Continuation)

Algorithm	Catfish			
	Human 157 MB		Human (salmon) 2.5 GB	
	Time (seconds)	Memory (MB)	Time (seconds)	Memory (MB)
SafePC	154.02	10.31	2413.97	21.16
ExtUnitigs	11.93	3.13	169.13	3.05
Safe&Comp	14.75	3.06	233.25	3.34
Greedy	69.74	5.93	1114.12	11.95

#### 5.4.1. Experimental results on the Catfish Data set

Since the Catfish data set does not have the genomic coordinates of nodes (exons or pseudoexons), the evaluation is based only on nodes.

**FIG. 6.**
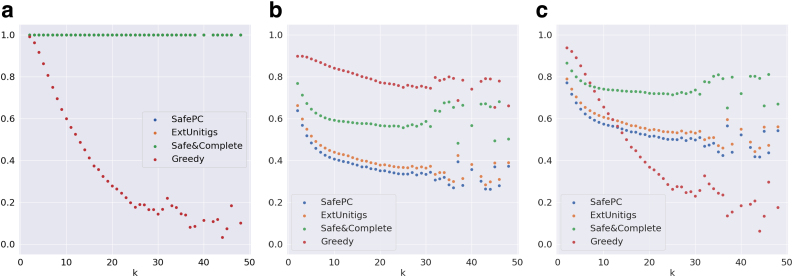
Evaluation metrics on graphs distributed by *k* for the Catfish data set. **(a)** Weighted precision, **(b)** maximum coverage, and **(c)** F-score.

**Remark 3.** *The results on the Catfish data set (Fig. 6) do not match the inferences from Section 5.4 exactly*. *The primary differences and expected reasons for this are as follows:*

*1. Base versus node computations for metrics: When considering the genomic content that is predicted (i.e., bases), Safe&Comp outperforms Greedy with respect to F-score over all graphs, as seen in Table 1. Because the Catfish data set has no base information, we can only report node information, but it is possible that the same patterns we observe in Reference-Sim with bases would hold for Catfish in terms of bases as well.*
*2. Ratio of simpler graphs: Catfish data sets are more skewed toward simpler graphs than the Reference-Sim data set. Table 1 shows that Reference-Sim has 32% of graphs with* k>10*, while Table 4 shows that the Catfish data set has only 2%. Since Greedy outperforms Safe&Comp on simpler graphs, it is better for the overall Catfish data sets having more simpler graphs*.

**Table 4.** Summary of Evaluation Metrics for the Catfish Data Set Without Funnels, Computed Relative to Nodes

Graphs	Algorithm	Maximum coverage	Weighted precision	F-score
k≥2 (100%)	SafePC	0.56	1.00	0.71
	ExtUnitigs	0.59	1.00	0.73
	Safe&Comp	0.71	1.00	0.82
	Greedy	0.89	0.92	0.89
2≤k≤10 (98%)	SafePC	0.57	1.00	0.71
	ExtUnitigs	0.59	1.00	0.74
	Safe&Comp	0.71	1.00	0.83
	Greedy	0.89	0.93	0.90
k>10 (2%)	SafePC	0.39	1.00	0.56
	ExtUnitigs	0.42	1.00	0.59
	Safe&Comp	0.58	1.00	0.74
	Greedy	0.82	0.49	0.56

#### 5.4.2. Experimental results including funnel instances

**FIG. 7.**
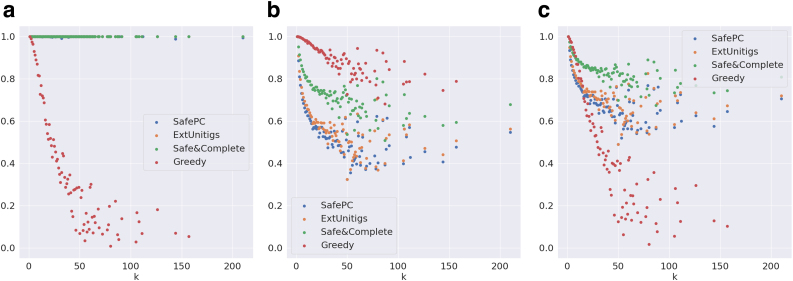
Evaluation metrics on graphs distributed by *k* for the complete (including funnels) Reference-Sim data set. **(a)** Weighted precision, **(b)** maximum coverage, and **(c)** F-score.

**FIG. 8.**
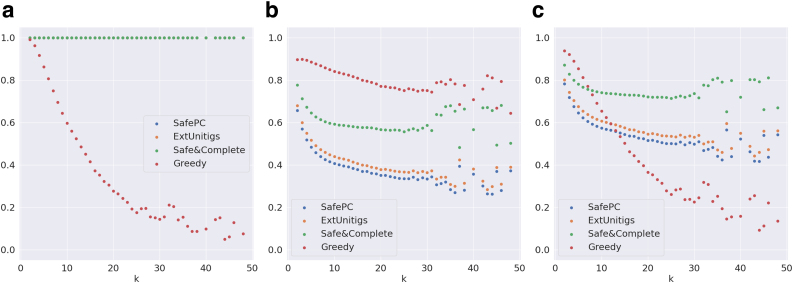
Evaluation metrics on graphs distributed by *k* for the complete (including funnels) Catfish data set. **(a)** Weighted precision, **(b)** maximum coverage, and **(c)** F-score.

 **Remark 4.** *The results when considering the complete data sets (including funnels) are diluted when compared with inferences from Section 5.4. In this case, we expect that the differences between the algorithms become less sharp, because all algorithms solve trivial (funnel) instances perfectly, which artificially increases the precision and coverage scores. This is confirmed by comparing Tables 1–5. Without funnel instances, we observe that the overall F-scores range between 0.66 and 0.9; whereas the range is from 0.82 to 0.95 when including them. A similar effect occurs for Catfish data in Tables 4 and 6. This is also visible from coverage and F-score metrics in Figures 7 and 8, which start from 100% even for safe paths, which is not the case in the corresponding figures without funnels*.

**Table 5.** Summary of Evaluation Metrics for the Complete (Including Funnels) Reference-Sim Data Set

Graphs	Algorithm	Maximum coverage	Weighted precision	F-score
k≥1 (100%)	SafePC	0.83	1.00	0.89
	ExtUnitigs	0.84	1.00	0.90
	Safe&Comp	0.91	1.00	0.95
	Greedy	0.99	0.91	0.93
1≤k≤10 (85%)	SafePC	0.87	1.00	0.92
	ExtUnitigs	0.88	1.00	0.93
	Safe&Comp	0.93	1.00	0.96
	Greedy	1.00	0.96	0.97
k>10 (15%)	SafePC	0.58	1.00	0.73
	ExtUnitigs	0.61	1.00	0.75
	Safe&Comp	0.76	1.00	0.86
	Greedy	0.95	0.61	0.70

**Table 6.** Summary of Evaluation Metrics for the Complete (Including Funnels) Catfish Data Set, Computed Relative to Nodes

Graphs	Algorithm	Maximum coverage	Weighted precision	F-score
k≥1 (100%)	SafePC	0.71	1.00	0.82
	ExtUnitigs	0.73	1.00	0.83
	Safe&Comp	0.78	1.00	0.87
	Greedy	0.87	0.96	0.91
1≤k≤10 (99%)	SafePC	0.72	1.00	0.82
	ExtUnitigs	0.73	1.00	0.83
	Safe&Comp	0.79	1.00	0.88
	Greedy	0.87	0.97	0.91
k>10 (1%)	SafePC	0.39	1.00	0.56
	ExtUnitigs	0.42	1.00	0.59
	Safe&Comp	0.58	1.00	0.74
	Greedy	0.82	0.48	0.56

## 6. CONCLUSION

We study the flow decomposition problem in DAGs under the Safe and Complete paradigm, which has applications in various domains, including the more prominent multiassembly of biological sequences. Previous work characterized such paths (and their generalizations) using a global criterion. Instead, we present a simpler characterization based on a more efficiently computable local criterion, which is directly adapted into an optimal verification algorithm, and a simple enumeration algorithm. Intuitively, it is a weighted adaptation of extended unitigs, which is a prominent approach for computing safe paths.

Through our experiments, we show that the safe and complete paths found by our algorithm outperform the popularly used greedy-width heuristic for RNA assembly instances with relatively complex graph instances, both on quality (F-score) and performance (running time and memory) parameters. On simple graphs, Greedy outperforms Safe&Comp, and Safe&Comp outperforms ExtUnitigs mildly (≈4−5%). However, on complex graphs, Safe&Comp outperforms Greedy significantly (≈20%) and ExtUnitigs appreciably (≈13%). While the Reference-Sim data set shows the overall dominance of Safe&Comp since complex graphs are appreciable (32%), Greedy dominates Safe&Comp in the Catfish data set since complex graphs are negligible (≈2%). Another significant reason for the dominance of Greedy over Safe&Comp on Catfish data sets is the absence of base information on nodes (see Section 5.4.1).

Hence, the importance of Safe&Comp algorithms increases with the increase in complex graph instances in the data set, and prominently when we consider information about the genetic information represented by each node. In terms of performance, Safe&Comp takes roughly 1.2−3× time than ExtUnitigs, both requiring equivalent memory. However, Greedy requires roughly 4−5× time and 1.3−3.6× memory than Safe&Comp. Overall, Safe&Comp performs significantly better than Greedy, without losing a lot over the trivial algorithms.

Despite the optimality of our characterization of safe and complete paths, the enumeration algorithm is not time optimal. In addition, the concise representation of the safe paths $P_{c}$ may not be optimal for some graphs as described in Section 4.3. Hence, for data sets with more complex graphs, there is a scope for improving the current enumeration algorithm and the concise representation in the future. Another interesting direction for an extension of this problem having practical significance is finding safe paths for those flow decompositions whose paths have a certain minimum weight threshold.

## Notes

^*^
Trancripts become paths of the corresponding splice graph.

^†^
This is not totally true in the case of safe paths for path covers since their algorithm reduces the solution space to increase coverage (Caceres et al., 2021; Section 3.1). However, their precision is close to 100% as shown by our experiments.

^‡^
We interpret the coverage as a measure of the *sensitivity* of the approaches.

^§^
If the constrained path covers required to cover the edges, then safe paths would be complete, which motivates further research on that approach.

^**^
The final expression level is obtained by multiplying 1000 to the sampled value, and then rounding this number to its nearest integer. If an expression level is zero, then this transcript is removed from the data set.

^††^

https://github.com/algbio/flow-decomposition-safety
